# Update Gender Affirming Care in Adolescents with Gender Incongruence – Evidence Review and Clinical Implications Considering new Guidelines in different European Countries

**DOI:** 10.1192/j.eurpsy.2025.137

**Published:** 2025-08-26

**Authors:** D. Pauli

**Affiliations:** Child and Adolescent Pychiatry, Psychiatric University Hospital Zurich, Zurich, Switzerland

## Abstract

**Abstract:**

Recently, there have been wide-ranging debates about gender affirming treatment for young people with gender incongruence. The ethical principle of self-determination of young people must be reconciled with a careful assessment and weighing up of the risks and benefits of medical treatment steps. The focus is on psychosocial support for young people struggling with gender incongruence in adolescence. The evidence for the effectiveness of medical interventions in adolescence is still limited. Nevertheless, there is a great need among affected adolescents. In a literature review and based on international debates, guidelines and treatment recommendations, a cautious and balanced approach to the question of the right support for adolescents with gender dysphoria is recommended.

**Disclosure of Interest:**

None Declared

